# Onboard visual micro-servoing on robotic surgery tools

**DOI:** 10.1038/s41378-025-00955-x

**Published:** 2025-05-29

**Authors:** Xu Chen, Michail E. Kiziroglou, Eric M. Yeatman

**Affiliations:** 1 Department of Electrical and Electronic Engineering, Imperial College London, London, UK; 2 Department of Industrial Engineering and Management, International Hellenic University, Thessaloniki, Greece; 3 College of Science and Engineering, University of Glasgow, Glasgow, UK

**Keywords:** Engineering, Electrical and electronic engineering

## Abstract

Precision motion actuation is a key technology for miniature medical robotics in a variety of applications, such as optical fibre-based diagnosis and intervention tools. Conventional inductive actuation mechanisms are challenging to scale down. Piezoelectric materials offer a scalable, precise, fast and high-force method but at a limited displacement range. In previous work, the combination of piezoelectric beams (benders) with compliant motion translation structures has been shown to be promising for robotic micro-actuation. In this paper, this approach is employed to implement a three degrees of freedom delta robot, suitable for catheter, diagnostic optical fibre and microsurgery tool manipulation. The fabrication process combines additive manufacturing, origami structuring and piezoelectric beam assembly. Closed-loop control is implemented using a new, on-board visual feedback concept. In contrast to typical optical motion systems, the fully internal visual feedback offers system compactness with precise and reliable camera-to-marker geometry definition. By employment of this method, a delta robot with motion accuracy of 7.5 μm, resolution of 10 μm and 8.1 μm precision is demonstrated. The robot is shown to follow a range of programmable trajectories under these specifications, and to compensate for externally applied forces typically expected during microsurgery manipulations. This is the first, to our knowledge, demonstration of micromotion control using internal visual feedback, and it opens up the way for high-resolution compact microrobots.

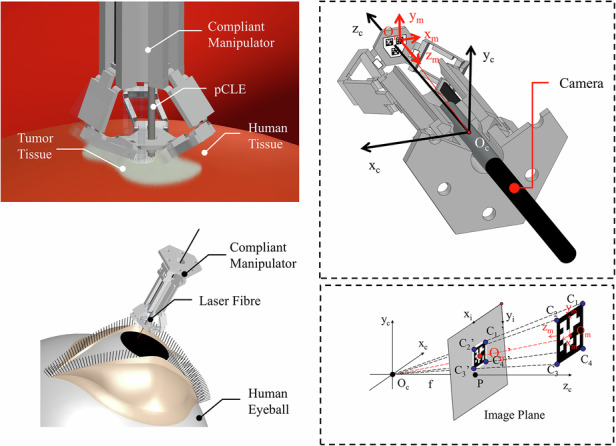

## Introduction

A robotic manipulation system for medical endomicroscopy is desirable to achieve lesion diagnosis and intervention in minimally invasive surgery^1^. It can steer a miniaturized optical fibre-based catheter to adjust the orientation and increase the effect area^2^. Such a manipulation system should have at least three degrees of freedom (DoF), high accuracy, high precision, and high power density actuation. Parallel manipulators have closed kinematic chain, which can provide more stability, a larger load-to-weight ratio, higher natural frequencies, and higher accuracy over traditional serial ones.^3,4^. However, parallel manipulators are difficult to miniaturise due to their joint and linkage assembly and driving systems. A promising method for implementing miniature manipulators is the employment of flexure-based compliant mechanisms^5^. Such mechanisms also offer the advantages of avoiding assembly and joint backlash. Also, piezoelectric beams provide fast and accurate motion actuation and a high load-to-weight ratio. Therefore, the combination of piezoelectric beams with compliant parallel structures can produce compact, high-precision and response speed motion^6^.

Miniature piezoelectric parallel manipulators have been studied widely in academia and industry in recent years. Lin et al. proposed a micromanipulator consisting of a hybrid planar flexure-based mechanism and piezoelectric stack actuators^7^. Suzuki et al. proposed an origami-inspired manipulator for teleoperated microsurgery based on a remote centre-of-motion structure^8^. These manipulators are actuated by piezoelectric linear actuators. Zhang et al. innovated a microneedle manipulator with four cross-arranged piezoelectric bimorph beams. It cooperates with the deflections of the beams to drive three DoF motion^9^. McClintock et al. proposed a milliDelta manipulator for vibration cancellation^10^. Kalafat et al. presented a novel origami-inspired delta mechanism^11^.

The delta robot architecture is particularly interesting for surgical and medical diagnosis tools due to inherent plane stability and 3 DoF control. An implementation combining flexure bending, compliant structure motion amplification and piezoelectric actuation can offer significant benefits in terms of accuracy, motion reproducibility and scalability to sub-millimetre dimensions^12^. Such implementations have been reported, demonstrating remarkable accuracy in open-loop control operation^10,13^. To enhance the suitability of these systems for medical operation applications, a compact closed-loop control mechanism is highly desirable, to improve motion repeatability and reliability, and suppress or account for external interference, for example, from gravity, electromagnetic field and haptic forces. Such feedback is often provided to robotic mechanisms by accelerometer sensors installed on the tip, hinge strain or force sensors or by motion tracking using an external camera^14,15^. The first method is based on advanced dead-reckoning positioning algorithms, with which, however, position drift accumulation is difficult to compensate for. Strain or force sensors provide an indirect position prediction, require considerable wiring across moving robot parts and are prone to noise. On the other hand, visual robotic feedback is routinely implemented using an external camera, limiting the device portability and compactness.

This paper introduces an onboard (self-contained) visual servoing system, implemented on an origami-inspired compliant delta micro-robot designed for robotic surgery manipulation tools^16^. The proposed approach combines the accuracy, reliability and simplicity of high-performance camera-based visual feedback, with compactness and independence from components installed in the environment, essential properties for portable systems. The key novelty point of this work is that it introduces micromotion control by an onboard visual feedback system. While a variety of systems employ external cameras or external position markings for visual feedback, this is the first time that a fully onboard visual feedback method has been proposed or implemented, to the knowledge of the authors. The method offers unique advantages in compactness, autonomy and reliability, as the microrobot is able to track and correct its own motion, without external sensing systems. It reduces dependence on environmental conditions and sight obstacles. It also doesn’t require an active component on the moving effector, lifting wiring size and environmental condition limitations. Overall, the internal visual feedback concept introduced in this paper has several key advantages over the current micromotion control state-of-art techniques, including:

- Visual feedback is a direct way of measuring relative position. Other methods, including strain or force sensing, are indirect and rely on complex models, which are time-consuming and restricted by the amount of measurement points^17^. They also require wiring which complicates the design and maintenance procedures, reduces system reliability and is often impractical or challenging to bridge across moving platforms.
- Commercial imaging chips offer very high precision, huge flexibility, high reliability, low power consumption, and very low cost compared with individual sensors.
- The visual method is essentially impervious to electrical or magnetic noise, whereas these are a major problem in force and strain sensing (e.g., piezo), especially in a confined geometry^18^.
- Internal visual feedback can provide wireless position control to electronically passive robotic tips, which can be beneficial for harsh, enclosed, sterile or biologically sensitive tip working areas.
- Internal visual feedback combines the benefits of conventional external visual feedback with compactness, portability, optical path reliability and stability^19^ as well as the possibility of engineering the visual link for 3D translational and 3D rotational self-orientation.

The system presented here consists of three parts: the micro-robot, a visual feedback system based on the robust AprilTag visual fiducial system^20,21^, and a servoing algorithm. In the second section, the actuated compliant structure is described and analysed, including a functional analysis and the fabrication method. The onboard visual servoing method is introduced in the third section, with a description of its implementation on the compliant micro-robot. Motion performance characterization experiments and corresponding results are presented in the fourth section. Discussion of the results and conclusions are presented in the fourth and fifth subections, respectively.

## The actuated compliant structure

**Fig. 1:**
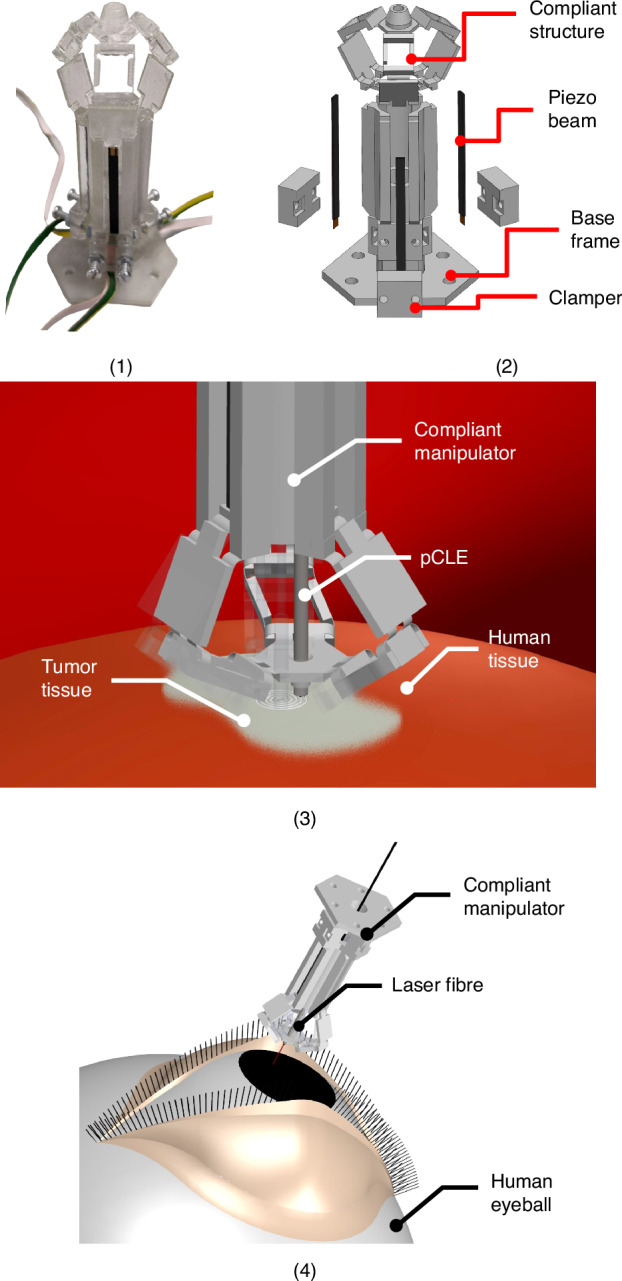
**1** Photo of the compliant micro-robot. **2** Exploded view of the delta micro-robot. **3** Cell-level intraoperative sensing. **4** Laser surgery for lesion tissue resection

**Fig. 2:**
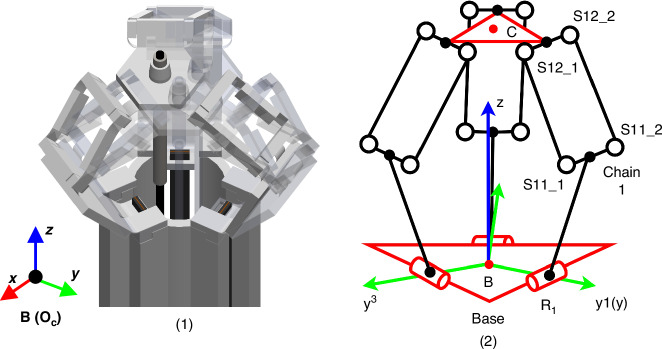
**1** Schematic diagram of the robot’s motion. **2** Corresponding kinematics model and symbols

**Fig. 3:**
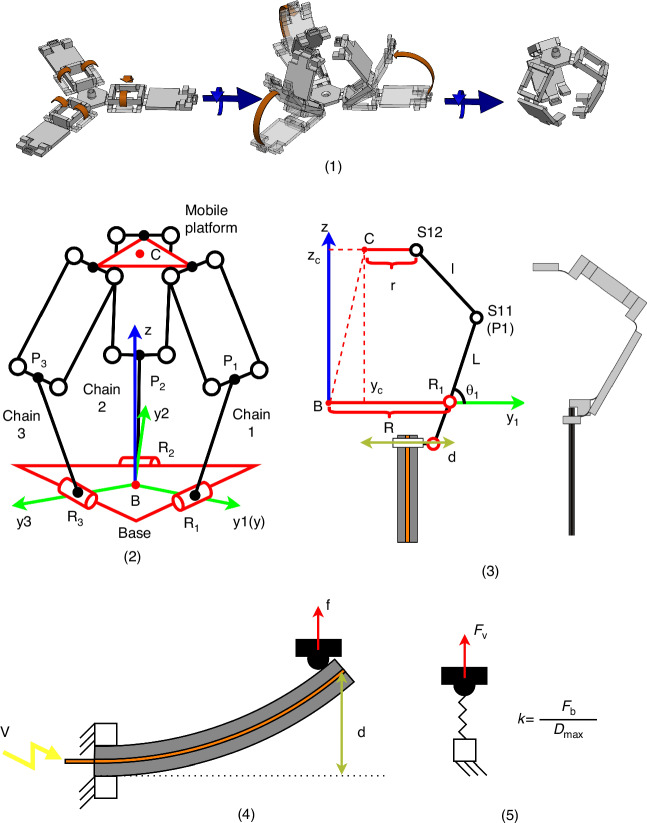
Schematic diagrams of **1** origami-like procedures of the compliant structure, **2** PRBM of the delta compliant structure. There are torsional springs applied at all the degrees of freedom of joints. **3** mechanical interface of the piezoelectric bender and the kinematic chain 1. **4** schematic of the piezoelectric beam under an external loading. **5** equivalent model of the piezoelectric beam under external loading

The micro-robot is based on a piezoelectrically-actuated compliant structure method introduced in refs. ^13,16^. It is fabricated by a combination of 3D-printing and origami assembly. A photo and an exploded view of the micro-robot are shown in Fig. 1 (1) and (2), respectively. The compliant structure and the three piezoelectric beams are fixed on a base frame, which is regarded as mechanical ground (stationary) in the analysis. The piezoelectric beams are clamped on the base frame and work as cantilevers with direct connection to the compliant structure (top head in Fig. 1 (2)), by insertion into mechanical slots, with their long axes parallel to the central axis of the 3D structure. The 3-DoF motion operation principle follows a delta-robot architecture and is illustrated in Fig. 2 (1). The platform is driven by three arms that are actuated at their lower end (R in Fig. 2) by piezoelectric beams. Each arm comprises two flexure-based rotational joints (S in Fig. 2) and a parallelogram frame with flexures. When an external voltage is applied they deflect and their deflections actuate a three DoF motion to the compliant structure platform, as shown in Fig. 1 (3). Small piezoelectric displacements at R are translated into larger x, y, z motion of the platform, through the combined effect of the three arms, while restricting rotation. The corresponding pseudo-rigid body model (PRBM) is shown in Fig. 3 (2). A detailed kinematic analysis of this delta-robot can be found in ref. ^13^. The transformation from electrical control signals to the top stage motion is performed using a quasi-static electromechanical (EM) model of the complete system^22^. Schematic diagrams of indicative target applications are shown in Fig. 1 (4) and (5).

### A. Design and Fabrication of the Compliant Structure

The actuation mechanism of the micro-robot comprises two essential components: an origami-inspired structure, and three piezoelectric beams. The EM modelling of the robot also comprises a compliant structure and a piezoelectric actuation part. The compliant structure follows a delta robot architecture actuated at three revolute joints. By replacing all joints with blade flexures and adding suitable mechanical interfaces for installing piezoelectric beam actuators, the conventional delta robot is transformed into a flexure-based parallel mechanism^5^. The mechanical properties of the blade flexures largely determine both the motion capability and the stability of the micro-robot. In the proposed method they are fabricated along with the delta-robot structure in a single 3D printing process using a Connex3 Objet500 PolyJet 3D Printer^23^. The structure is printed in flat form and subsequently transformed into 3D form by an origami-like procedure. This method allows all the blade flexures to be printed in parallel with the printing platform, improving material homogeneity which is critical for flexure functionality. An acrylate-based polymer compound of proprietary composition named VeroClear™^23^ was used as the printing material. A theoretical analysis and design parameter selection discussion of this robot architecture has been published in ref. ^16^. The flexure design was based on a combined theoretical and experimental study presented in ref. ^24^.

**Fig. 4:**
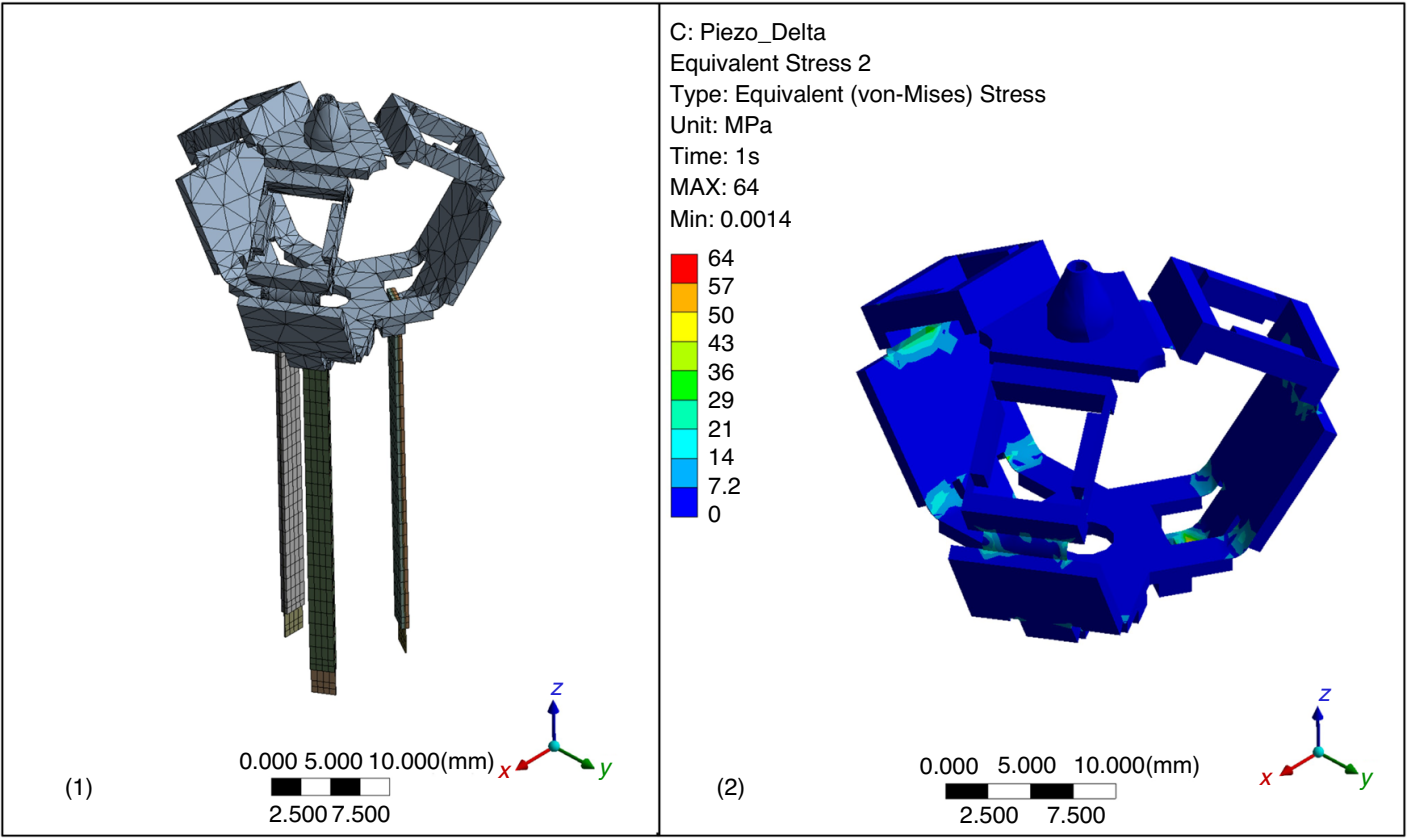
Diagrams during FEA study on ANSYS. **1**: Meshing plot. **2**: Equivalent (von-Mises) stress plot of the compliant stress under 150 V input

A computer-assisted design (CAD) model of the origami- procedure is shown in Fig. 3 (1). The robot base is formed by gluing the three parallelogram mechanisms, which will be folded into three-dimensional shapes. Subsequently, the three kinematic chains are unfolded and assembled onto the robot base. Further details of this fabrication method, dimensions and material properties can be found in ref. ^16^. The specifications of the piezoelectric beams are those of type 6 device as reported in ref. ^25^. Finite element analysis (FEA) was conducted based on ANSYS Workbench 2023 R2^26^ to support the reliability of the piezo-actuated compliant structure (Fig. 4 (1)). The maximum stress is 64 MPa at 150 V supplied (Fig. 4 (2)). This is within the material strength specifications as provided in ref. ^16^, demonstrating a reliable expected stationary operation.

### B. Compliant structure modelling

A pseudo-rigid-body model (PRBM) was used to build the quasi-static model of the compliant structure^5^. It is an approximation method for modelling the large deflection of small-length-flexure-based mechanisms. Each flexure joint is represented by a revolute joint coupled with a torsional spring. The equation of the spring stiffness is:

1 $$K=\frac{{(EI)}_{t}}{{l}_{t}}$$

Here, *E* is the Young’s modulus of the material. *I* is the second moment of inertia of the bending section and *l* is the flexure length. The subscript *t* indicates that the values correspond to bending with respect to the thickness of the flexure. The position of each revolute-spring joint is at the geometrical centre of the blade flexure. The compliant structure was originally a delta robot mechanical system transformed by replacing all joints with blade flexures. The forward kinematic equations of the PRBM is the same as those of a delta robot, as shown in Fig. 3 (2) and (3)^16,27^. The modelling of a compliant parallel mechanism should combine the kinematic model of the delta robot and the flexures’ torque balance. The quasi-static motion of the compliant structure can be derived by solving the balance of kinematics and torque balances.

### C. Piezoelectric beam modelling

The modelling of a piezoelectric beam involves the combination of the inverse piezoelectric effect and the strain mismatch in the bimorph/trimorph structure employed^28,29^. Here, a simple linear model is employed which is an acceptable approximation given the small deflection angle and the fact that only the tangential component of the beam tip displacement is transferred to the compliant structure through the interface slot. To apply this model, the following assumptions are made: 1. The piezoelectric beams are electrode-end fixed. 2. The external loading on the beams is in the bending direction and concentrated at their tips. 3. Hysteresis, thermal properties and creep and drift effects are negligible at the motion speeds involved. 4. Only static and quasi-static motions are considered. Then, the equation of the external force of the piezoelectric beam is^30,31^:

2 $$f={F}_{V}+{F}_{s},\,{\rm{where}}:\,{{F}}_{V}=\frac{{F}_{b}}{{V}_{{\max }}}V,{F}_{s}=-\frac{{F}_{b}}{{D}_{{\max }}}d$$

Here, d indicates the tip displacement, V is the applied voltage, and *f* is the external force. *V*_*max*_, *F*_*b*_ and *D*_*max*_ are the maximum operation voltage, blocking force and the maximum deflection of the piezoelectric beams, which are properties of the beams. These are also depicted in Fig. 3 (4). Equation 2 shows that the interaction force f between a piezoelectric beam and the forced object is equal to the sum of two forces, which are the piezoelectric force *F*_*V*_ and the mechanical stiffness force *F*_*s*_. *F*_*V*_ is an external force proportional to the applied voltage, and has the same direction as the deflection. It represents the force from the piezoelectric material. *F*_*s*_ is a passive spring force also applied along the deflection direction, which respresents the compliance of the piezoelectric beam. One side of the spring is linked to the forced object-bender interface, while the other is fixed to the ground. Its equilibrium position is the zero-force contact position. Its stiffness is *k*_*s*_ = *F*_*b*_/*D*_*max*_. The corresponding model is shown in Fig. 3 (5). Using this model to represent each of the three piezoelectric beams of the robot, in combination with the model in subection B, an overall electromechanical model is obtained. In this way, the effective spring constant at the piezoelectric beam tips can be analyzed as one part of the compliant structure, and *F*_*V*_ can be regarded as a constant-direction external force.

### D. Combined modelling and verification

In order to form an overall EM model, a combined use of the Simulink Simscape Multibody^32^ toolbox and ANSYS-based FEA was employed. Simulink toolbox can combine both models presented in subections B and C of this section, mimicking the motion of the overall actuation mechanism. Spring stiffnesses as well as initial positions and stress, can be defined at the joints. This is very useful as some of the flexures are, in general, not at their equilibrium states because they include internal stress introduced by the origami procedure at the overall robot equilibrium position. This effect would be complicated to model by equation analysis or FEA, but it can be easily included to the Simulink model as biasing parameters. More details on this approach is presented in ref. ^22^. The effectiveness of the model was verified by a ANSYS-based FEA as well. The simulation results of Simulink and FEA indicate that the micro-robot is a quasi-linear system, whose behaviour is not severely affected by the internal stress caused by origami folding. The combined three DoF actuation follows the superposition principle^16^. Based on this model, a voltage-to-displacement vector translation matrix was derived, using external optical motion tracking. The matrix values were identified by training a ten-layer neural network through the Levenberg–Marquardt algorithm. The reason for not using linear regression was to avoid control variations due to printing and assembly imperfections.

## The method of onboard tag-based visual servoing

The neural network EM model presented in the former section can be used directly for both open-loop and closed-loop position control of the micro-robot. However, an open-loop motion control strategy makes it difficult to account for structural parameter variations, printing and assembly imperfections, hysteresis,small-scale material creep, drift and thermal effects. In addition, external effects such as field and haptic forces need to be compensated for a repeatable motion control strategy. Therefore, a closed-loop control mechanism is better for medical and other applications. The broad availability of high-performance, low-cost cameras has resulted in the adoption of visual feedback as a motion control mechanism in a wide range of applications. In most such cases, cameras are positioned in a location external to the controlled system. Such a technique would be challenging to implement on portable micro-robots due to the system overall size and the small scale motion. For this reason, we propose the use of an onboard camera to create a fully self-contained visual feedback system. This is the first time, to the knowledge of the authors, that an internal camera is employed for micromotion control, and offers a range of advantages as outlined in the introduction of the paper.

### A. Calculation of coordinates

**Fig. 5:**
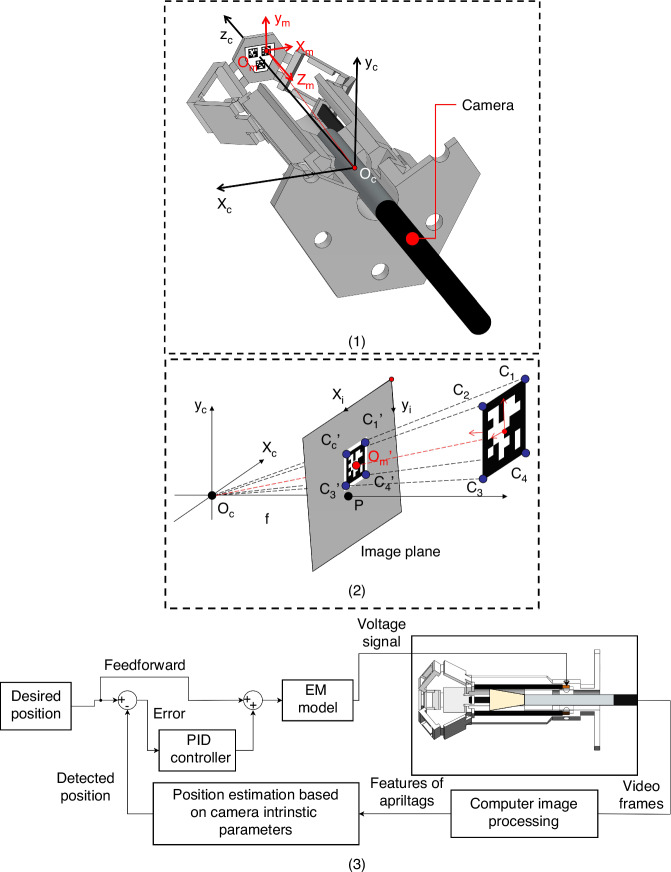
Working principle of the AprilTag-based motion sensing system with a single camera. **1** Camera captures the AprilTag on the mobile platform and estimates the transfer matrix from the markers’ local coordinate system (*x*_*m*_, *y*_*m*_, *z*_*m*_) to the camera coordinate system (*x*_*c*_, *y*_*c*_, *z*_*c*_). **2** Positions of corners (${C}_{1}^{\text{'}}$, ${C}_{2}^{\text{'}}$, ${C}_{3}^{\text{'}}$, ${C}_{4}^{\text{'}}$) and centre (${O}_{m}^{\text{'}}$) of the AprilTag in the image plane are recognised and used to drive the pose of the AprilTag in the camera coordinate system. The camera coordinate system is considered to be fixed, and the motion are measured with respect to it. **3** Workflow chart of the visual servoing algorithm

Visual servoing systems are mainly classified into three types: image-based visual servoing (IBVS)^33^, position/pose-based visual servoing (PBVS) and hybrid^15^. A simple PBVS system is developed as a demonstration of the proposed approach. AprilTag is a widely used visual fiducial system for robotics^20,21^. The software can compute the precise position and orientation of targets with respect to the camera coordinate system by detecting specific groups of markers attached to them. The markers are two-dimensional barcodes. The architecture of onboard visual feedback is illustrated in Fig. 5 (1). It provides a 6 × 1 array of parameters describing the translational and rotational displacement of the targets. For the technology demonstration presented in this paper, only the translation data are exploited.

The tracking principle of the visual servoing system is illustrated in Fig. 5 (2). The camera coordinate system is defined as (*x*_*c*_, *y*_*c*_, *z*_*c*_). This is also the robot’s coordinate system as the camera location is fixed. Each captured image (grey plane in Fig. 5 (2)) can be addressed by a two-dimensional pixel coordinate system (*x*_*i*_, *y*_*i*_). The corners and centre of the AprilTag square pattern are identified as ${C}_{1}^{\text{'}}$, ${C}_{2}^{\text{'}}$, ${C}_{3}^{\text{'}}$, ${C}_{4}^{\text{'}}$ and ${O}_{m}^{\text{'}}$ in the (*x*_*i*_, *y*_*i*_) pixel addressing system using an image pattern recognition routine. These coordinates can be translated into the real position coordinates (*x*_*c*_, *y*_*c*_, *z*_*c*_), using the pixel-to-distance ratios in the x and y directions, *f*_*x*_, *f*_*y*_ respectively, as well as offset parameters *c*_*x*_ and *c*_*y*_. These four mapping parameters are specified by image calibration using MATLAB routines^34^ and printed templates provided by AprilTag^20,21^. In summary, the marker position in the image (X_*i*_, *Y*_*i*_), in pixel coordinates, is translated to its real position coordinates (*X*, *Y*, *Z*) by Eq. 3.

3 $$Z\left[\begin{array}{c}{X}_{i}\\ {Y}_{i}\\ 1\end{array}\right]=\left[\begin{array}{llll}{f}_{x} & 0 & {c}_{x} & 0\\ 0 & {f}_{y} & {c}_{y} & 0\\ 0 & 0 & 1 & 0\end{array}\right]\left[\begin{array}{c}X\\ Y\\ Z\\ 1\end{array}\right]$$

### B. Physical Architecture

The camera is inserted upward through the bottom of the base frame and monitors the three AprilTags attached beneath the mobile platform, as shown in Fig. 5 (1). The tags remain parallel to the X-Y plane of the camera’s inner coordinates during motion because of the in-plane stage confinement of the delta robot architecture^27,35^. The camera chosen is a *ϕ*3.9 mm Borescope Inspection Snake Camera^36^. It can generate thirty 640 × 480 frames per second. This rate is slower than the response speed capability of the piezoelectric driving circuitry (in the 8 kHz range) and of the digital controller. It is the main limiting factor of the control capability of the micro-robot. To avoid further delay in the feedback loop, the video capturing, image processing, and voltage-supplying tasks are apportioned to separate programming processes in Python.

### C. Visual servoing algorithm

A flow chart of the closed-loop control logic is presented in Fig. 5 (3). The target position point is updated in real time to follow a given trajectory and velocity profile. In parallel, the position is monitored by the visual feedback system. The error between the desired position and the measured position is derived and fed to a proportional – integral – derivative (PID) controller. A feedforward control term of the desired position is added to the PID controller output as a predicted control value to increase response speed and stability. In addition, this feedforward term reduces the influence of high-frequency noise, which is hard to filter, due to the illumination, especially in the Z direction^37^. Then, the sum of feedback and feedforward terms are provided to the EM model to calculate the required voltage supply values for the desired motion.

## Experimental results

To validate the effectiveness of the visual servoing control, open and closed-loop motion control experiments were conducted. The first is a circular trajectory tracking experiment for controlling the micro-robot with its onboard visual servoing system. The second regards the characterization of motion resolution. The third demonstrates the performance of the visual closed-loop control in compensating for trajectory deviations due to external forces such as gravity. The setup and results are presented in the following sections.

### A. Experimental Setup

**Fig. 6:**
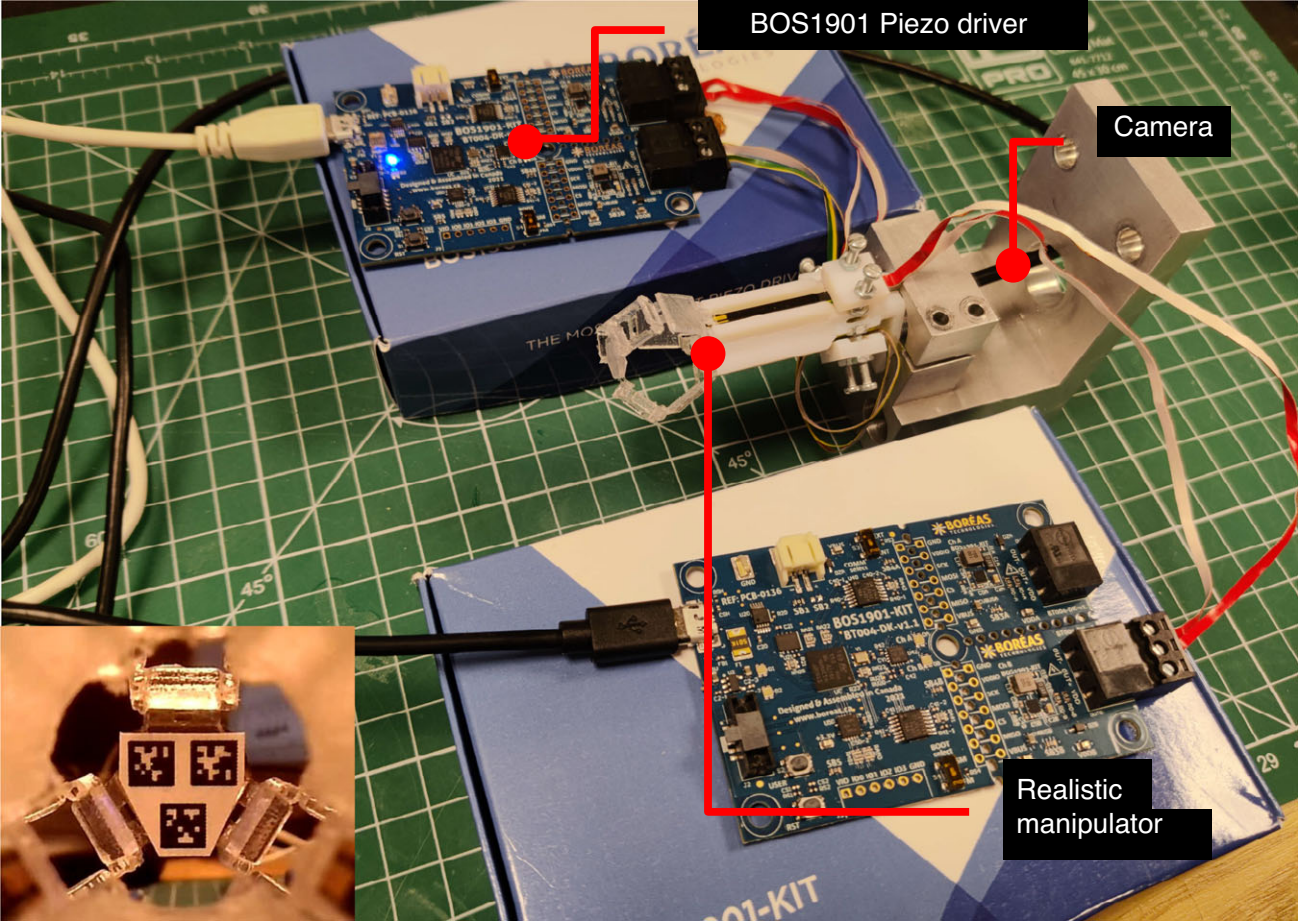
Photo of the overall experimental setup and an example frame shot by the camera (left-bottom)

A photograph of the experimental setup is shown in Fig. 6. An indicative camera view is also shown in the inset. The micro-robot was bolted on a fixed base. The main axis of the micro-robot is parallel to the table surface. The y-axis of the AprilTag markers at the equilibrium position is perpendicular to the table and upward, so that the gravity is towards the negative y-axis. Trajectory following and resolution estimation experiments were conducted using this setup. The coefficients of the PID controller were manually tuned to suitable values, namely *P* = 0.8, *I* = 0.0025, and *D* = 0.1. For gravity disturbance recovery experiments, a mass-negligible basket containing weights was hung on the mobile platform. The details of the experiments are as follows.

### B. Trajectory Following

First, the working envelope of the platform was measured experimentally to be 0.618 mm^3^ under ± 95 V voltage input. This is overlapped with the theoretical envelope of 0.552 mm^3^ predicted using the FEM simulations of Fig. 4^22^. In this comparison, nominal values for all dimensions, displacements and material properties were used, without any fitting.

**Fig. 7:**
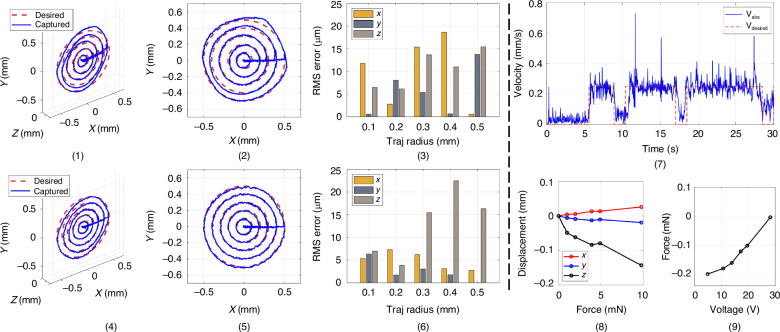
Circular trajectory following data plot by open-loop and closed-loop control and error plots. Open-loop: **1**–**3**. Closed-loop: **4**–**6**. **1**, **4** Overview of the trajectories. **2**, **5** X-Y plan view. **3**, **6** RMS error analysis plot. The reference (or desired trajectories) are plotted in red dashed lines in (**1**, **2**, **4**, **5**). The actual (or detected) trajectories are in blue. The error plots show the absolute 2D errors of the experimentally measured trajectory. **7** Absolute speed profile for the closed loop circular trajectory following. **8** Passive z-axis displacement caused by the axial loadings without actuation. **9** Measured z-axis actuation force vs voltage applied symmetrically to the three piezoelectric beams

Subsequently, open-loop-controlled motion and closed-loop-controlled motion of the micro-robot are compared in terms of trajectory following performance. Circular trajectories with a radius from 0.1 to 0.5 mm were mapped with an absolute speed value of 0.25 mm/s. The trajectories are all 2D concentric circles on the X-Y plane of the global coordinate system. Errors in X, Y and Z directions between measured and desired positions were compared. The results of the trajectory followed by open-loop and closed-loop control are shown in Fig. 7. For both cases, the errors of the X and Y directions are relatively stable, but that in the Z direction increases with the radius of trajectories. To characterise the performance of the system in following a predefined trajectory, the trajectory-following accuracy was measured as the root–mean–square (RMS) error of deviation between measured and desired positions in the x, y and z directions for a complete desired trajectory. The trajectory distinction resolution is defined as the minimum distinguishable distance between adjacent trajectories. The trajectory following precision is defined as the RMS deviation error among multiple trajectory runs.

The RMS error in Fig. 7 (6) does not exceed 7.5 µm. Overall, a reduced X and Y direction error is demonstrated by closed-loop control. The Z direction error is similar for both cases. This is attributed to the low visual tracking resolution in the Z direction. It is also observed that the X and Y errors are adequately low in both cases. This demonstrates the high resolution and repeatability of piezoelectric-actuated compliant structures, even without feedback control. In the larger radius trajectories, the relatively large error in the Z direction in both open and closed-loop operation is due to the large corresponding applied voltage required as the robot approaches its motion range boundaries. Indeed, voltage instability was observed near the 95 V maximum voltage specification of the piezoelectric beam drivers used, which was observed to be regulated by the visual servoing system. Each radius circle was followed five times to calculate the RMS precision of the micro robot compared with the average trajectory. The precision was found to be 8.1 µm.

An experimental characterization of the three-dimensional speed, loading mechanical response and force actuation can be found in^13,38^. Indicatively, the measured average speed profile for the closed-loop circular trajectory following is presented in Fig. 7 (7). The time interval between different circles is 1.5 second. The 3D displacement response of the platform as a function of z-axis load force is shown in Fig. 7 (8). Finally, z-axis actuation force measurements as a function of voltage applied symmetrically to the three piezoelectric beams are shown in Fig. 7 (9).

### C. Resolution estimation

**Fig. 8:**
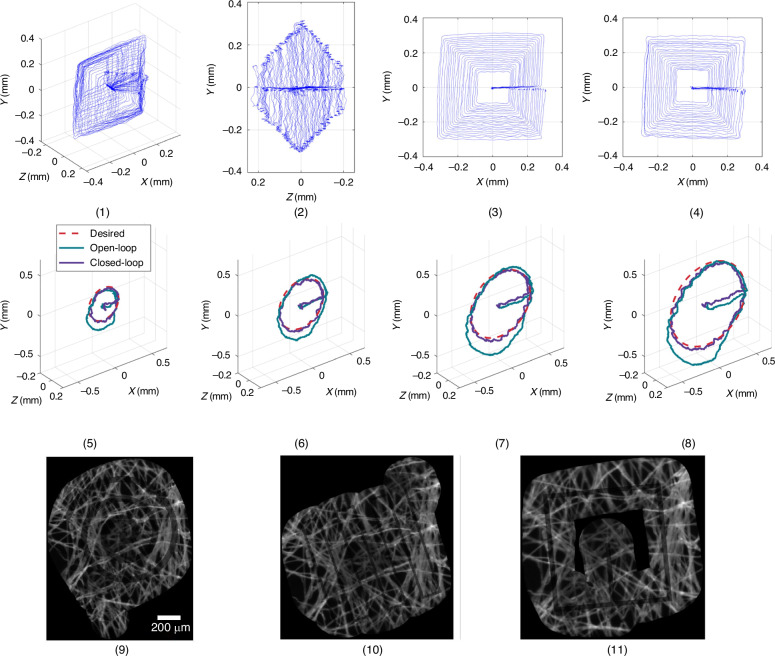
Diagrams (**1**)-(**4**) are trajectory data plot for the resolution estimation. **1** Overview of the trajectories. **2** Y-Z plane view. **3** Negative Z direction view. **4** Positive Z direction view. **5**–**8** are the results of the gravity disturbance recovery experiments with different trajectory radii. The weight of the mass is 200 mg. **5** 0.2 mm. **6** 0.3 mm. **7** 0.4 mm. **8** 0.5 mm. Stitched pCLE scans of dyed lens tissue using a spiral (**9**), a raster (**10**) and a square (**11**) trajectory sequence

The motion resolution of the micro-robot was estimated by determining the minimum distance between two adjacent trajectories of the micro-robot, which serves to distinguish them during measurement. The absolute speed value was 0.8 mm/s. A square trajectory shape with relatively higher scanning speed was selected to allow direct resolution analysis in the x and y directions. The Z dimension of the 2D plane in which the squares were placed increased by the same step. Considering the working space^16,22^, the attainable square side length decreased with Z on either side (higher or lower) of the equilibrium plane. This resulted in the 3D shape of all square trajectories being similar to a combination of two pyramids mirrored about the X-Y plane. In Fig. 8, the resolution evaluation measurements are presented. Multiple experiments with different displacement steps have been carried out. The minimal step that results in a clear distinction of adjacent trajectories was found to be 10 μm. The double-pyramid shapes with a step of 10 μm are shown in Fig. 8 (1). The Z direction view is shown in Fig. 8 (2). The demonstrated Z-axis resolution is lower. This is attributed to the limited visual tracking resolution in the Z direction. These results demonstrate a trajectory distinction resolution of 10 μm. The single position resolution provided by the piezoelectric beams is below this value but beyond the detection capability of the onboard optical position measuring system used.

The trajectory following performance demonstrated in this paper is comparable to the state-of-the-art, such as the milli-delta introduced by Suzuki and Wood^8^ (indicatively, 26 μm precision, a novel origami-inspired delta mechanism put forward by Kalafat et al.^11^ and the Micro-X4 proposed by Feng et al.^39^ (indicatively 10 μm targeted accuracy). In addition, the proposed architecture is unique in employing onboard visual feedback, which permits a compact system architecture. Finally, it offers simpler fabrication procedures, and it is not bench-based, allowing application to catheters and other portable surgical tools.

### D. Gravity disturbance recovery

These experiments aim to validate that the closed-loop control can handle the disturbance of constant external loading.

To reveal the effectiveness of the onboard visual system, circular 2D trajectories with 0.2 to 0.5 mm radii are used for comparison. Experimental results of circular motion with four different radius values are separately plotted in Fig. 7 (5)–(8). The gravity direction is along the negative direction of the Y-axis. In all measurements, the 200 mg weight first caused an initial shift of the equilibrium point along the gravity direction by -0.055 mm. The X-direction width of these trajectories shrank about 15%. In the positive Y direction, the maximum value shows a shift similar to that of the equilibrium point. In the negative Y direction, the trajectories shifted by 0.2 mm. Closed-loop control trajectories restored the shift of the equilibrium point as well as the circular trajectory shape. These experiments demonstrate the advantage of using the visual servoing system to compensate for external force effects.

## Conclusion

An onboard visual servoing method is proposed for medical micro-robots. A prototype system was implemented and evaluated on a piezoelectric beam-actuated compliant delta micro-robot. Such systems require compact onboard feedback to overcome piezoelectric hysteresis effects, and to compensate for external disturbing factors such as gravity and haptic forces. The method employed AprilTag visual markers, an endoscope camera, custom digital control software implementing kinetostatic analysis, actuator modelling, visual tracking and parallel-differential feedback control algorithms. Based on the kinematic analysis of the manipulator, the visual servoing system provides motion feedback through image-based feature capturing, which increases accuracy and also compensates for the effect of external loading and the hysteresis phenomena of piezoelectric materials. Experimental results demonstrate controlled motion with 7.5μm accuracy, 8.1μmprecision and 10μm resolution motion. Gravity recovery experiments also validate the ability to correct for external disturbances.

A limitation of the demonstrator prototype presented is that the low frame rate (30 fps) of the probe-camera introduces significant delay to the visual feedback control speed, in the order of 33 ms. This reduces the feedback reaction speed and introduces significant control lag, which in turn prevents high-speed response to external motion noise or tool-user tremor. The adoption of a higher speed camera would significantly improve the speed and dynamic stability of the microrobot. For example, the Omnivision OH08A endoscope camera^40^ could provide 60 fps at 4024 × 2180 pixel resolution, at a 7 mm × 4.5 mm package size. The adoption of such a camera would reduce the frame-rate delay to 17 ms, improving the dynamic stability and allowing compensation for low-frequency external noise or tremor. A second limitation is the lower z-axis trajectory-following accuracy due to the limited depth-tracking performance of the motion trackers used. Significant z-axis and overall accuracy is expected by employing enhanced visual depth tracking methods such as multiple tags, 3D geometrical markers, shadow tracking or combined 3D markers and projected patterns for depth and rotation angle tracking. In addition, the compactness of the onboard visual feedback concept introduced here allows for blocking external lighting interference, use of smaller and higher resolution markers as well as consistent and predictable light-marker and marker-camera lines of sight. Thereby, it is expected to improve accuracy and visual control consistency in real operating environments.

Scaled-down implementations are promising for integrating the proposed robotic concept into catheter diagnostic and surgical instruments. With the current fused deposition modelling and assembly method, scaling down by an overall dimensions factor of 3 is possible using commercially available cameras and piezoelectric beam devices. For an overall size below 2 cm^3^ the availability of camera and piezoelectric beam models becomes a limiting factor. Further work towards such small-scale implementations may employ design and development of bespoke beam and camera components as well as assisted assembly techniques such as using structural guides during folding. Overall, the combination of piezoelectric actuation with compliant structures and on-board visual feedback introduced in this paper is expected to enable portable micro-scale motion control of high precision, reliability, and compactness, suitable for catheter micro-surgery and biomedical diagnosis tools. As an indicative application, the robot was employed as a scanner for probe-based confocal endomicroscopy (pCLE) experiments. Indicative stitched images of a tissue using a spiral, a raster and a square trajectory sequence are shown in Fig. 8 (9), (10) and (11), respectively. For these measurements, a pCLE probe was installed through the microrobot axis and manipulated by the controlled motion of the top platform.

In conclusion, a new visual feedback method for micro-motion control is presented in this paper. The use of an internal camera allows precise definition and command of the optical link thereby offering several key advantages including high-resolution, repeatability, compactness, portability, reliability and electromagnetic noise immunity. It does not require specialized high-end precision components, as the camera microchip industry offers a range of mature, accessible, low-cost and high-performance devices that are easy to integrate. The experimental demonstration of performance enhancement by the proposed method presented is a key milestone towards compact, high-precision micro-robotic devices.
